# Supplementary material for: The accuracy of several pose estimation methods for 3D joint centre localisation
Source: Sci Rep. 2021 Oct 19;11:20673. doi: 10.1038/s41598-021-00212-x (PMC8526586; doi:10.1038/s41598-021-00212-x)
Supplement: Supplementary file 2 — Supplementary Information 1. [file 41598_2021_212_MOESM2_ESM.docx]

**SUPPLEMENTARY MATERTIALS**

**Supplementary Video Examples**

Supplementary videos examples are provided to demonstrate the outputs of our markerless motion capture pipeline. The videos with the suffix “*3D_RES.mp4*” provide examples of the final 3D reconstructed skeleton for walking, running and jumping overlaid in a select number of 2D fields of view. The joint centres from these results were compared with joint centres derived from marker-based motion capture. In all video examples, DeepLabCut derived results are rendered in red, OpenPose derived results are rendered in cyan and AlphaPose derived results are rendered in green.

The videos with the suffix “*2Dv3D.mp4*” provide examples of the 2D pose estimation results for select field of view and the final 3D joint centres. 2D results are rendered as circular markers whose external colour denotes the pose estimation method while their internal colour denotes the confidence value (between 0 and 1) returned for that marker. Confidence values are colour mapped where red indicates a low confidence value, yellow a moderate value and green a high confidence value. 3D joint centres are rendered as a vectors between joint centres.

The video DLC_RECONSTRUCTION_ROBUST_VS_NON.mp4 provides an example of our robust 3D reconstruction method (green lines) vs a standard triangulation-based reconstruction with no outlier detection/correction algorithm (magenta). Note that neither set of 3D trajectories has undergone smoothing or filtering in this example, hence the high-frequency noise in the results is more notable when compared to the final Kalman smoothed examples shown in other the videos provided.

**Pose Estimation Runtime Settings**

OpenPose, AlphaPose and DeepLabCut results were obtained by processing data on four graphics processing units (GPUs). These included two Nvidia Titan Xp, one Nvidia GeForce GTX 1080 Ti and one Nvidia GeForce GTX 2080 Ti with Cuda version 10.2.

OpenPose, AlphaPose and DeepLabCut were run with the following settings and model weights:

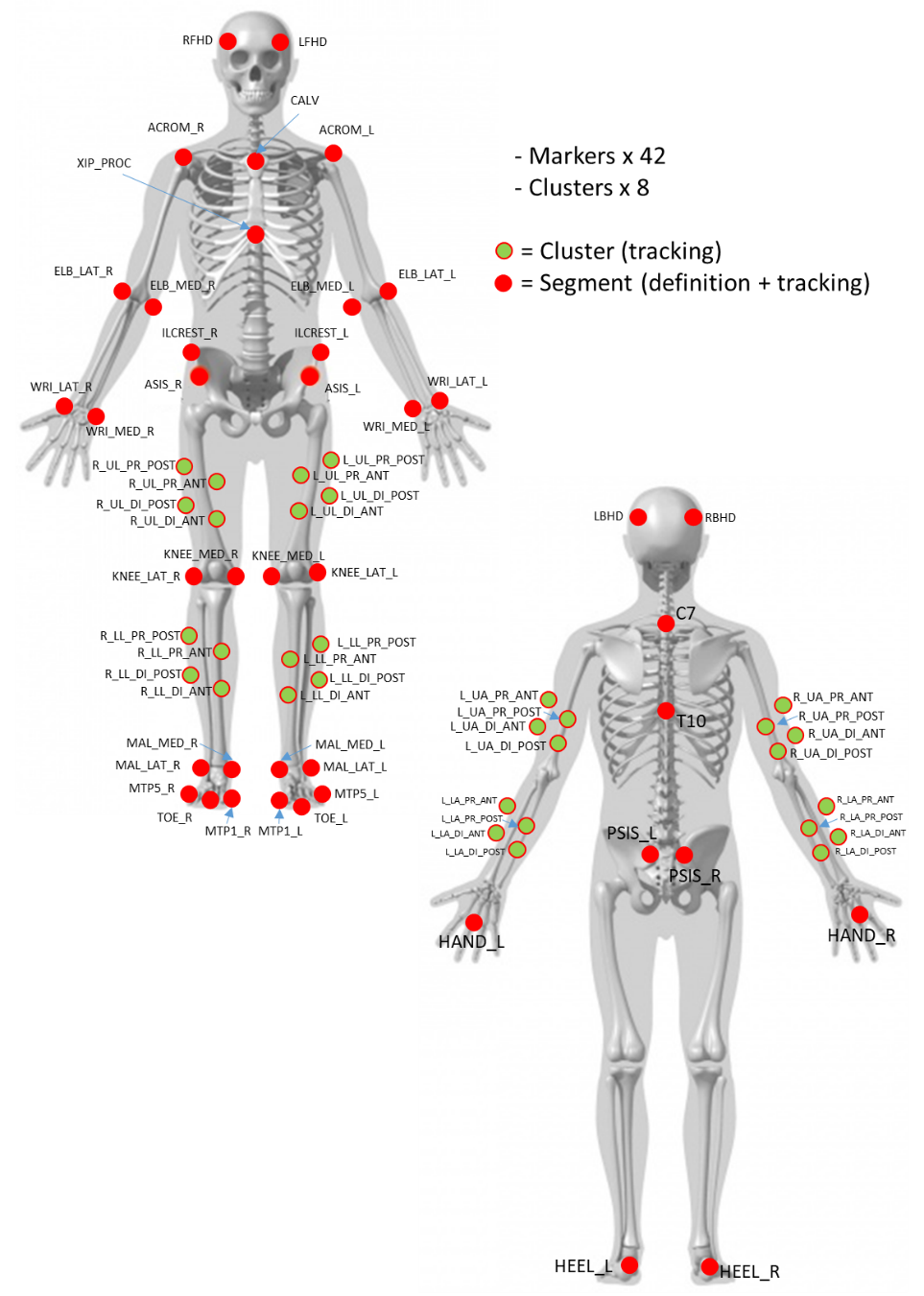


**Figure A1. Anatomical locations of markers (red dots) and clusters (green dots) on the body provide 6 DoF tracking of 14 body segments.**

**
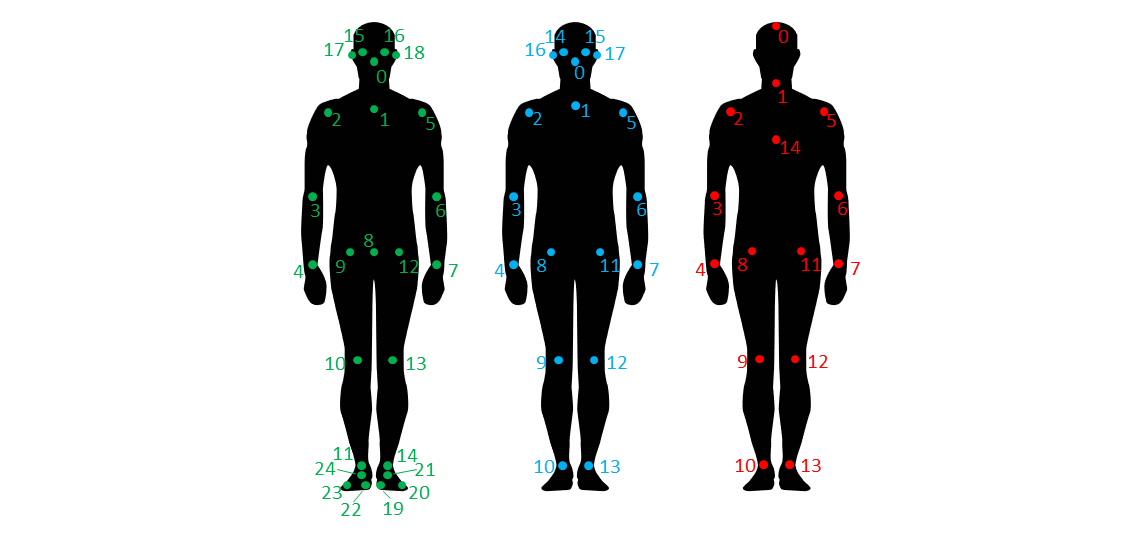
**

**Figure A2. Keypoint outputs for OpenPose (left), AlphaPose (centre) and DeepLabCut (right). Keypoint numbers corresponding to body landmarks are given in Table A1**.

**Table A1. Keypoint locations for each pose estimation method as described in the training datasets. Numbers correspond to the keypoint models shown in Figure A2.**

| OpenPose Landmark & Number | AlphaPose Landmark & Number | DeepLabCut Landmark & Number |
| --- | --- | --- |
| 0 - Nose | 0 - Nose | 0 - Head |
| 1 - Neck | 1 - Neck | 1 - Neck |
| 2 - Right Shoulder | 2 - Right Shoulder | 2 - Right Shoulder |
| 3 - Right Elbow | 3 - Right Elbow | 3 - Right Elbow |
| 4 - Right Wrist | 4 - Right Wrist | 4 - Right Wrist |
| 5 - Left Shoulder | 5 - Left Shoulder | 5 - Left Shoulder |
| 6 - Left Elbow | 6 - Left Elbow | 6 - Left Elbow |
| 7 - Left Wrist | 7 - Left Wrist | 7 - Left Wrist |
| 8 - Mid Hip | 8 - Right Hip | 8 - Right Hip |
| 9 - Right Hip | 9 - Right Knee | 9 - Right Knee |
| 10 - Right Knee | 10 - Right Ankle | 10 - Right Ankle |
| 11 - Right Ankle | 11 - Left Hip | 11 - Left Hip |
| 12 - Left Hip | 12 - Left Knee | 12 - Left Knee |
| 13 - Left Knee | 13 - Left Ankle | 13 - Left Ankle |
| 14 - Left Ankle | 14 - Right Eye | 14 - Chest |
| 15 - Right Eye | 15 - Left Eye |  |
| 16 - Left Eye | 16 - Right Ear |  |
| 17 - Right Ear | 17 - Left Ear |  |
| 18 - Left Ear |  |  |
| 19 - Right Big Toe |  |  |
| 20 - Right Small Toe |  |  |
| 21 - Right Heel |  |  |
| 22 - Left Big Toe |  |  |
| 23 - Left Small Toe |  |  |
| 24 - Left Heel |  |  |


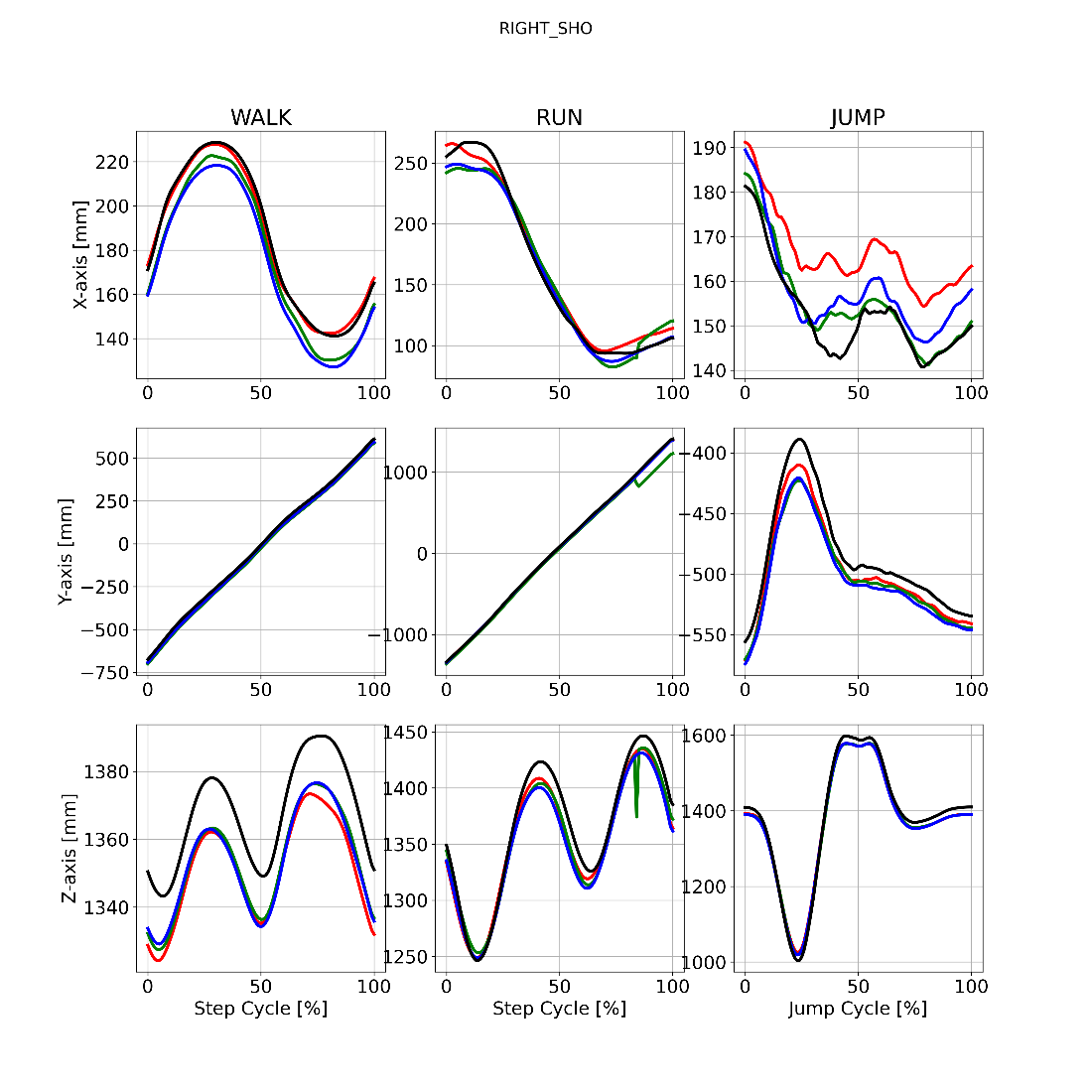


**Figure A3. Example mean right shoulder joint centre trajectories for a single participant during walking (left), running (centre) and jumping (right). Marker based trajectories (black), OpenPose (green), AlphaPose (blue) and DeepLabCut (red).**


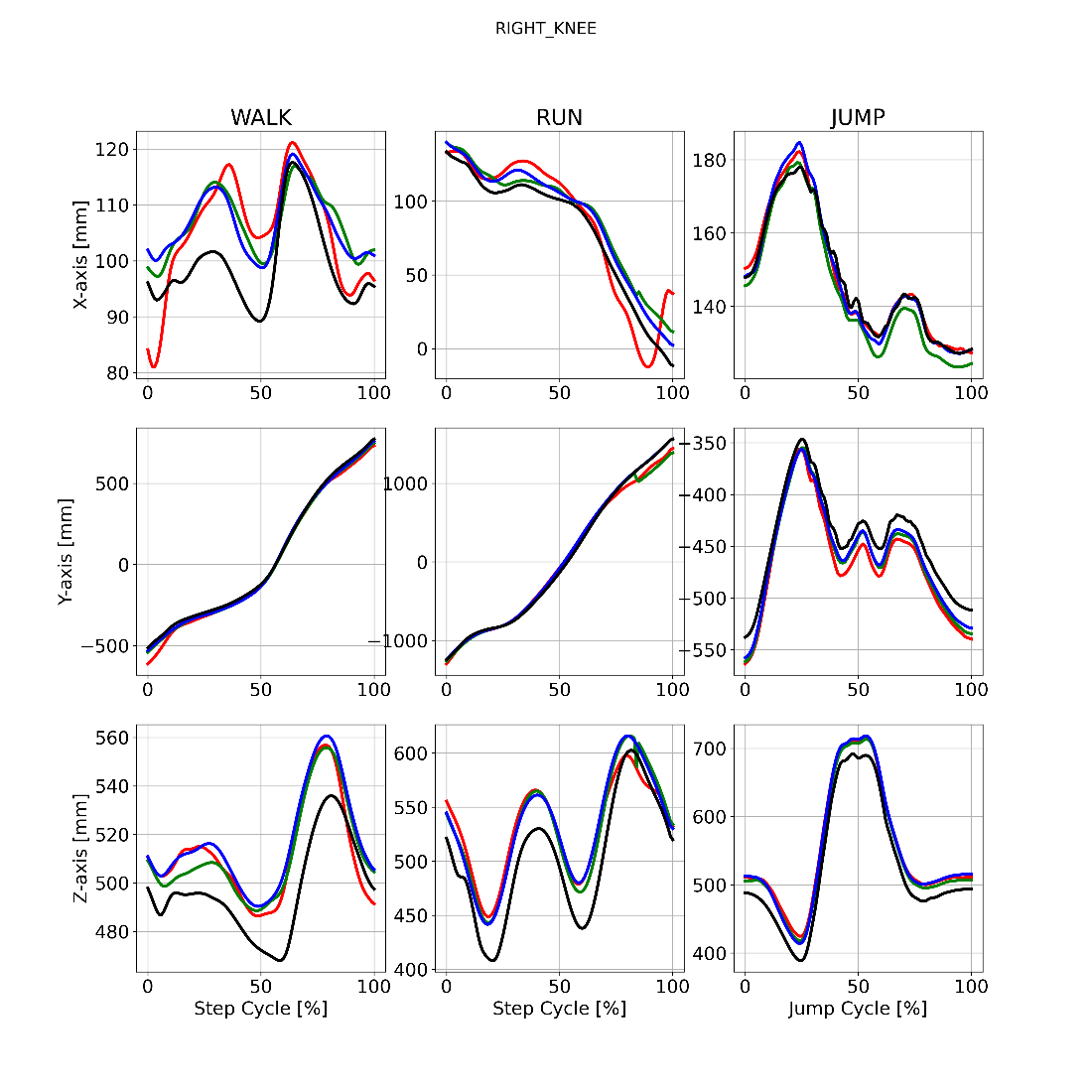


**Figure A4. Example mean right knee joint centre trajectories for a single participant during walking (left), running (centre) and jumping (right). Marker based trajectories (black), OpenPose (green), AlphaPose (blue) and DeepLabCut (red).**


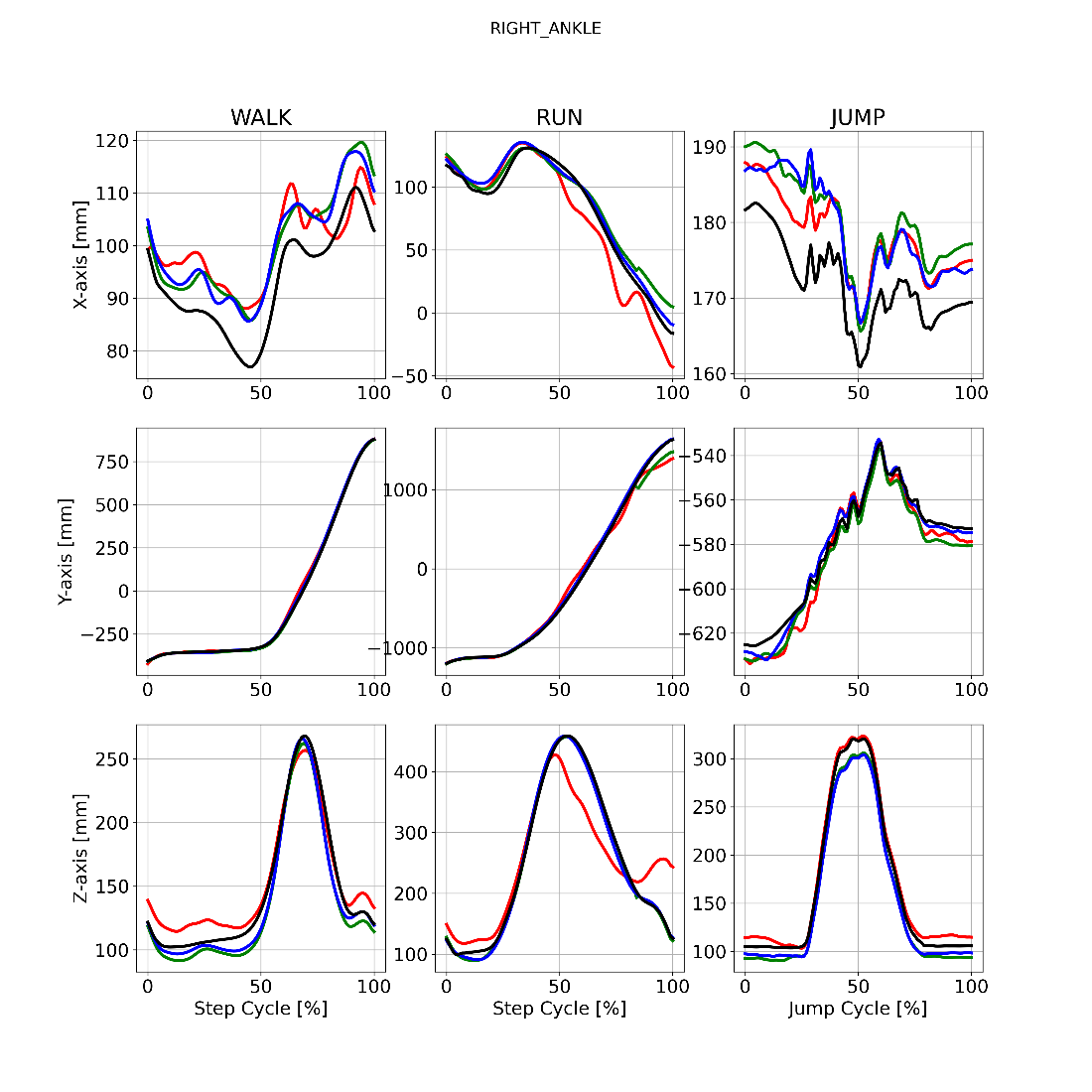


**Figure A5. Example mean right ankle joint centre trajectories for a single participant during walking (left), running (centre) and jumping (right). Marker based trajectories (black), OpenPose (green), AlphaPose (blue) and DeepLabCut (red).**


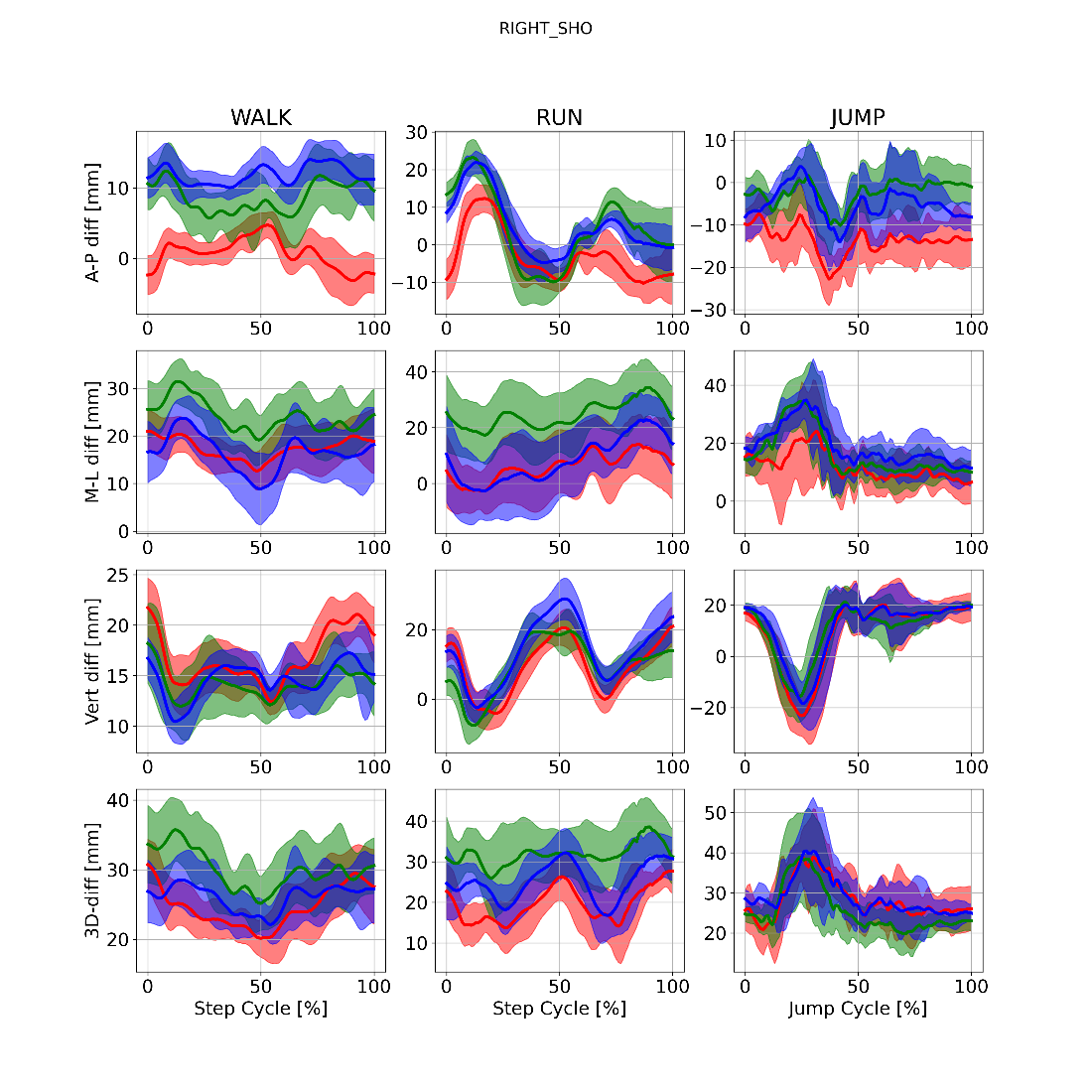


**Figure A6. Example mean (± SD) differences between marker based and markerless trajectories for the right shoulder joint centre trajectories of a single participant during walking (left), running (centre) and jumping (right). Marker based trajectories (black), OpenPose (green), AlphaPose (blue) and DeepLabCut (red). Row 0 - anterior-posterior differences. Row 1 - medial-lateral. Row 2 - superior–inferior differences. Row 3 – 3D Euclidean differences.**


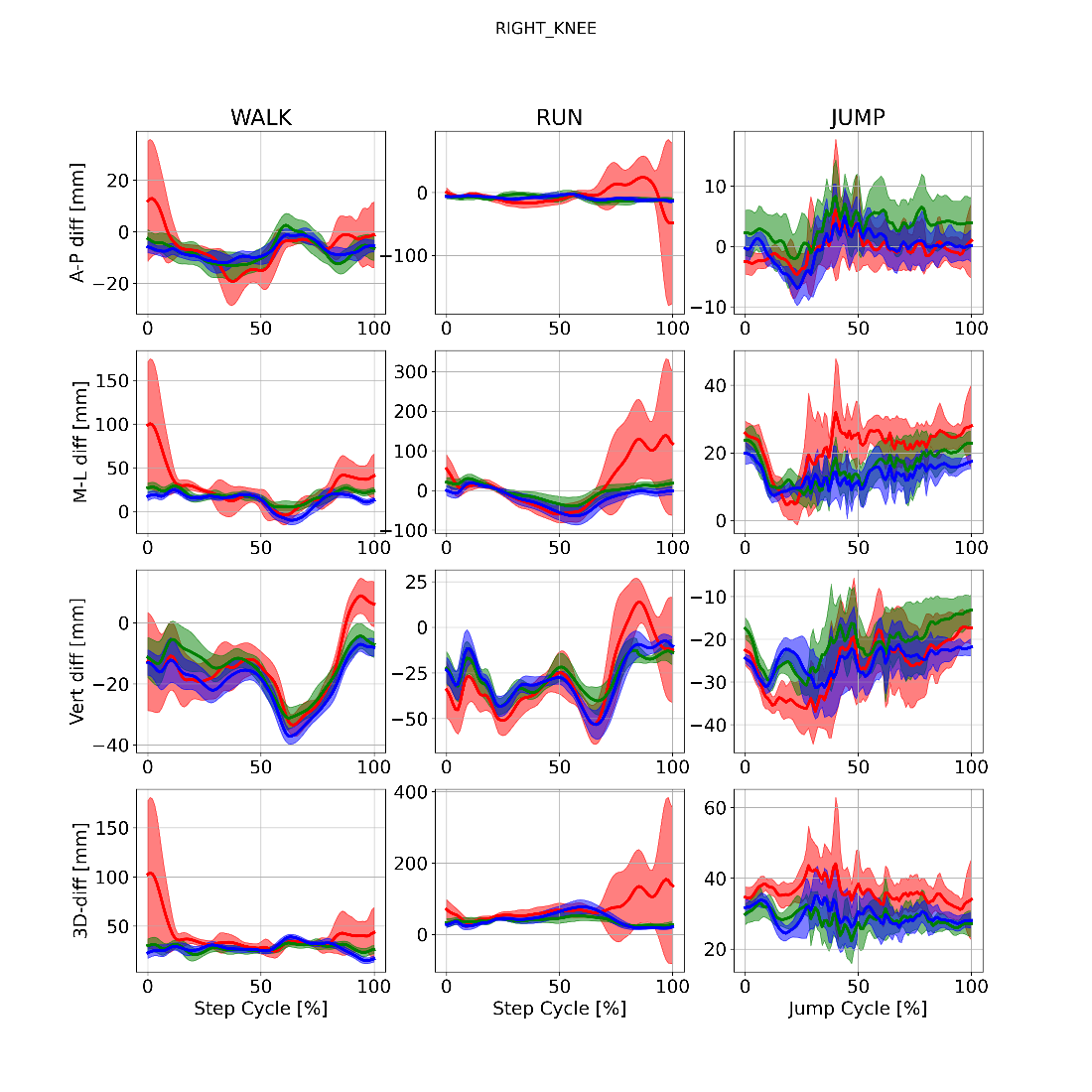


**Figure A7. Example mean (± SD) differences between marker based and markerless trajectories for the right knee joint centre trajectories of a single participant during walking (left), running (centre) and jumping (right). Marker based trajectories (black), OpenPose (green), AlphaPose (blue) and DeepLabCut (red). Row 0 - anterior-posterior differences. Row 1 - medial-lateral. Row 2 - superior–inferior differences. Row 3 – 3D Euclidean differences.**


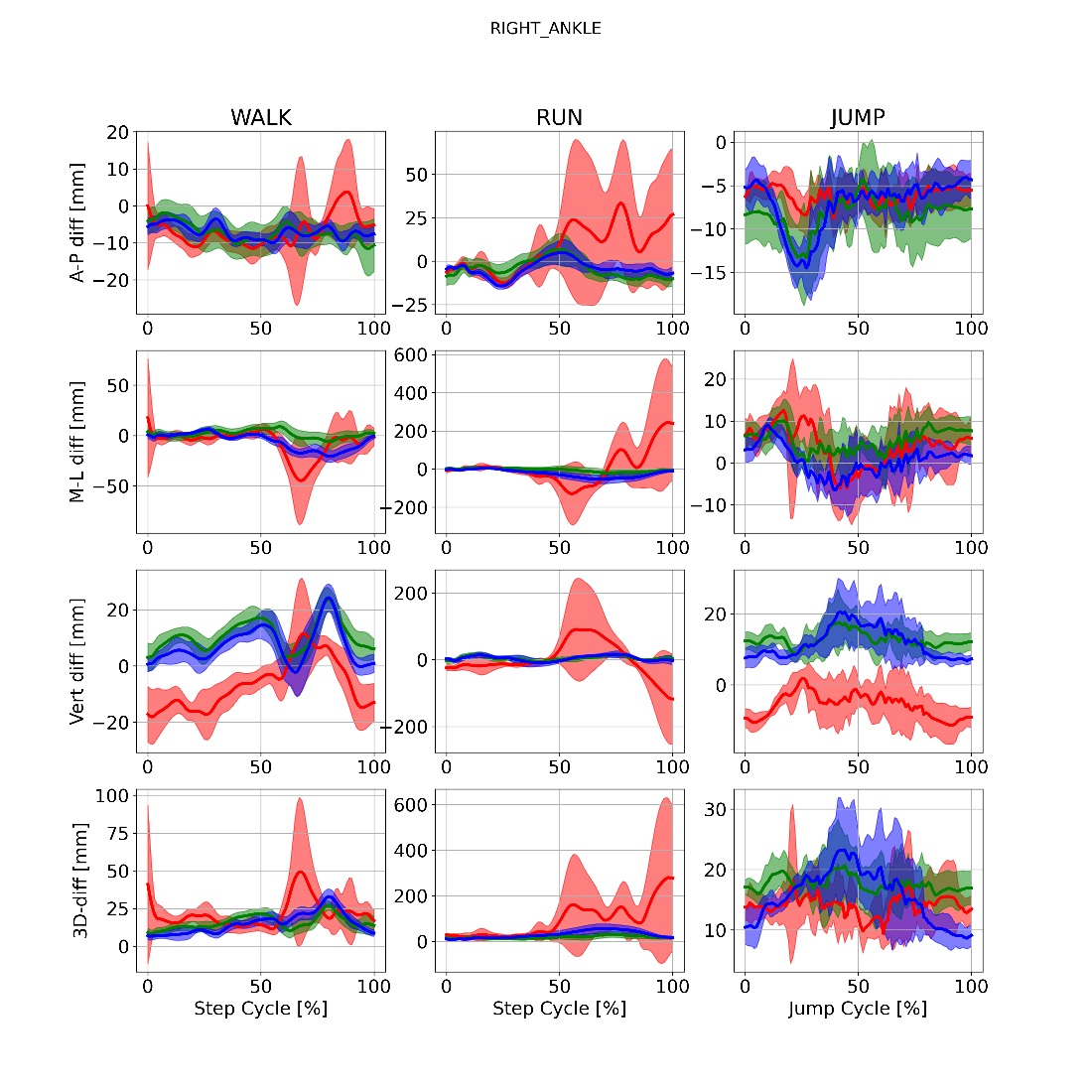


**Figure A8. Example mean (± SD) differences between marker based and markerless trajectories for the right ankle joint centre trajectories of a single participant during walking (left), running (centre) and jumping (right). Marker based trajectories (black), OpenPose (green), AlphaPose (blue) and DeepLabCut (red). Row 0 - anterior-posterior differences. Row 1 - medial-lateral. Row 2 - superior–inferior differences. Row 3 – 3D Euclidean differences.**


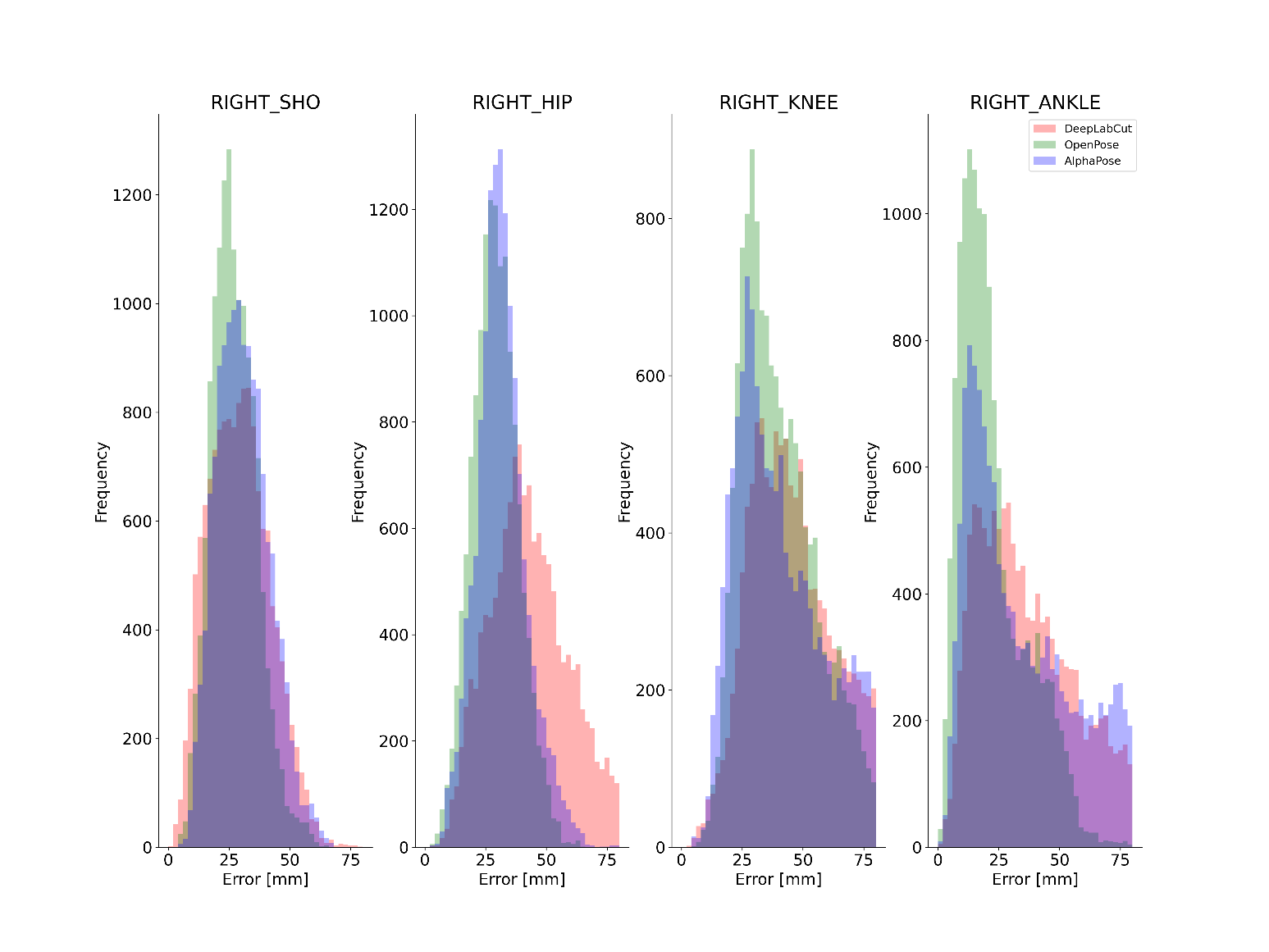


**Figure A9. Error distributions during running for each pose estimation method at the right shoulder (left), hip (centre-left), knee (centre-right) and ankle (right) joint centres.**


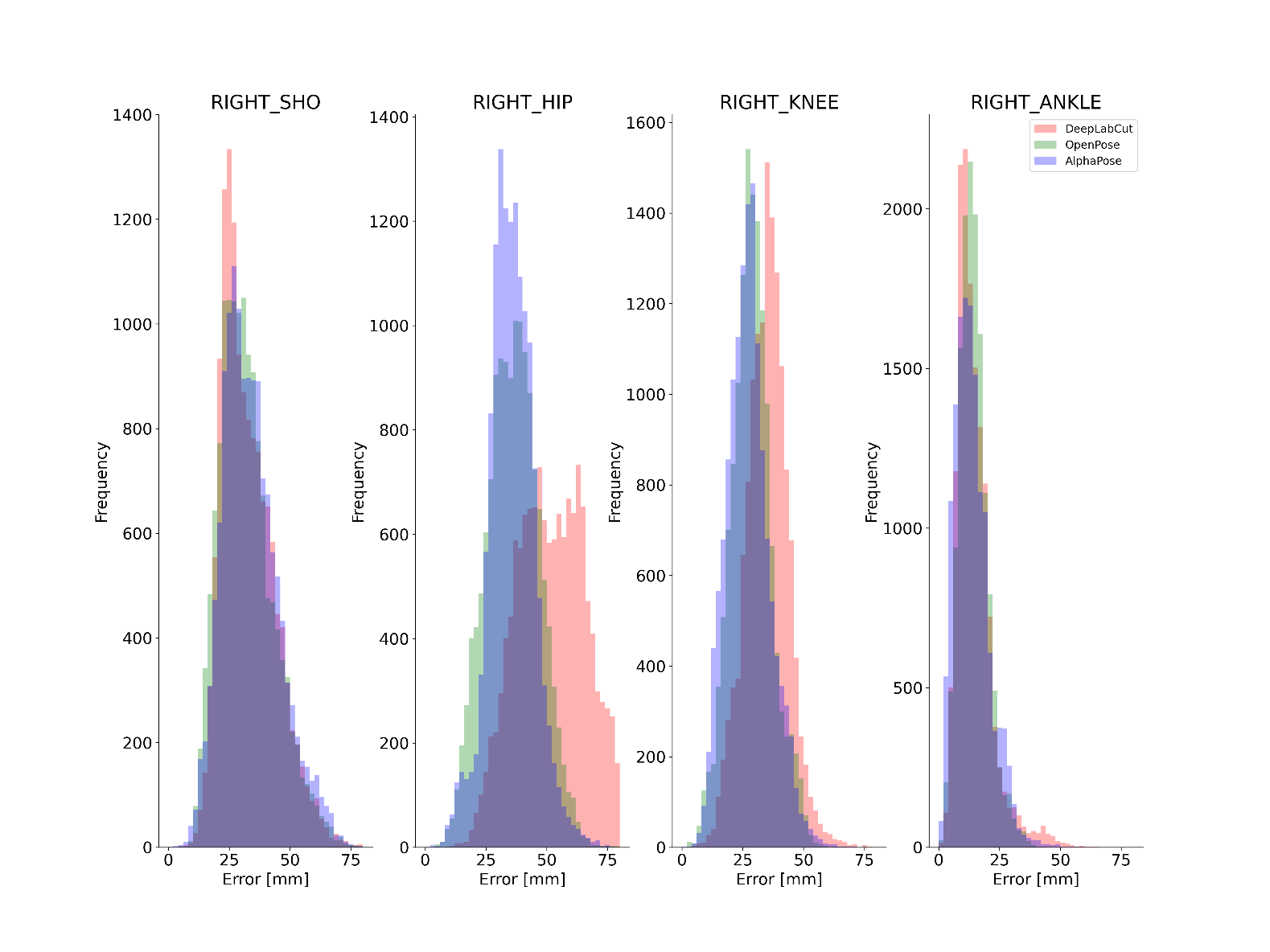


**Figure A10. Error distributions during jumping for each pose estimation method at the right shoulder (left), hip (centre-left), knee (centre-right) and ankle (right) joint centres.**


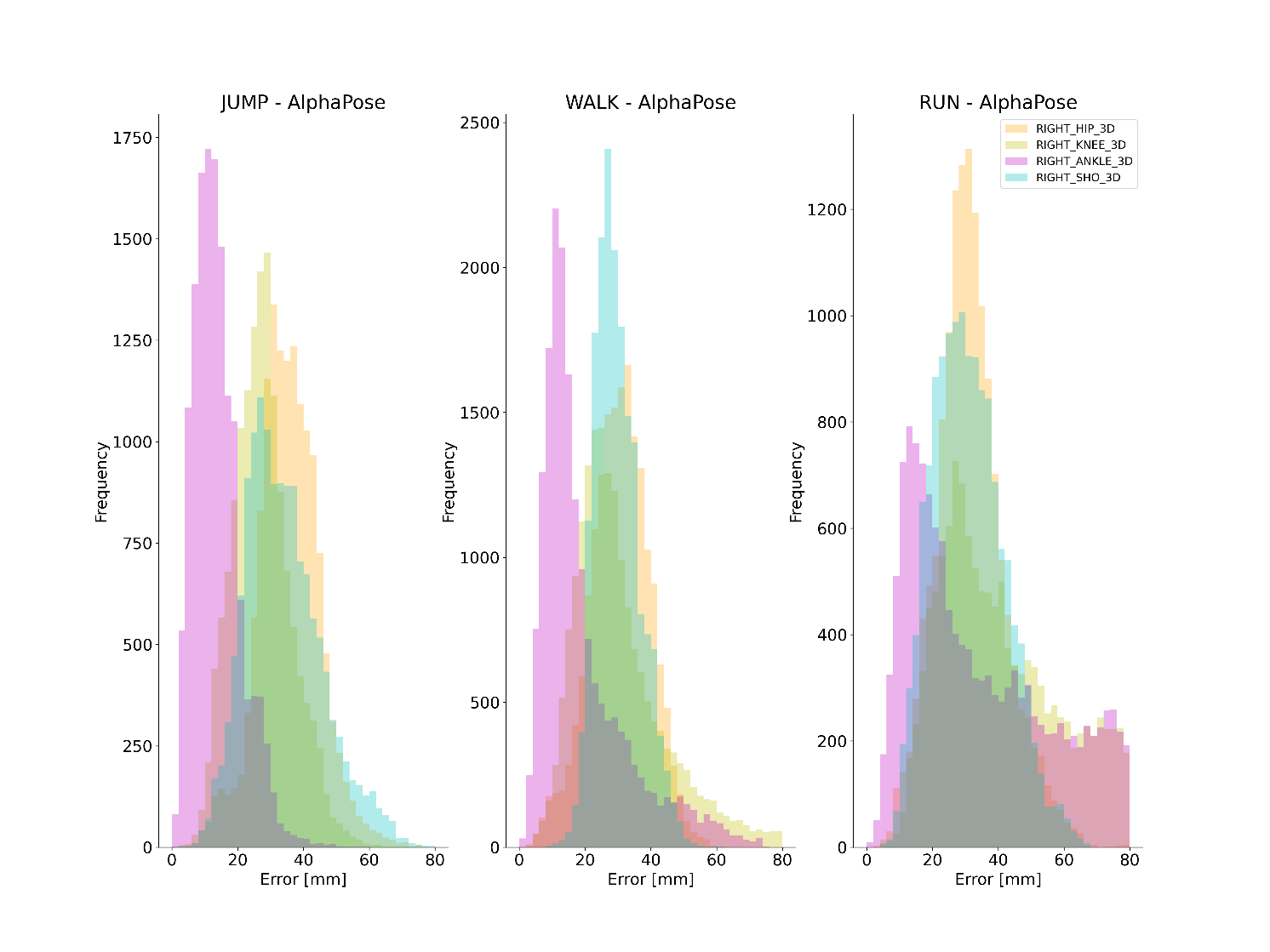


**Figure A11. Error distributions for AlphaPose at the right shoulder, hip, knee and ankle joint centres for jumping (left), walking (centre) and running (right).**


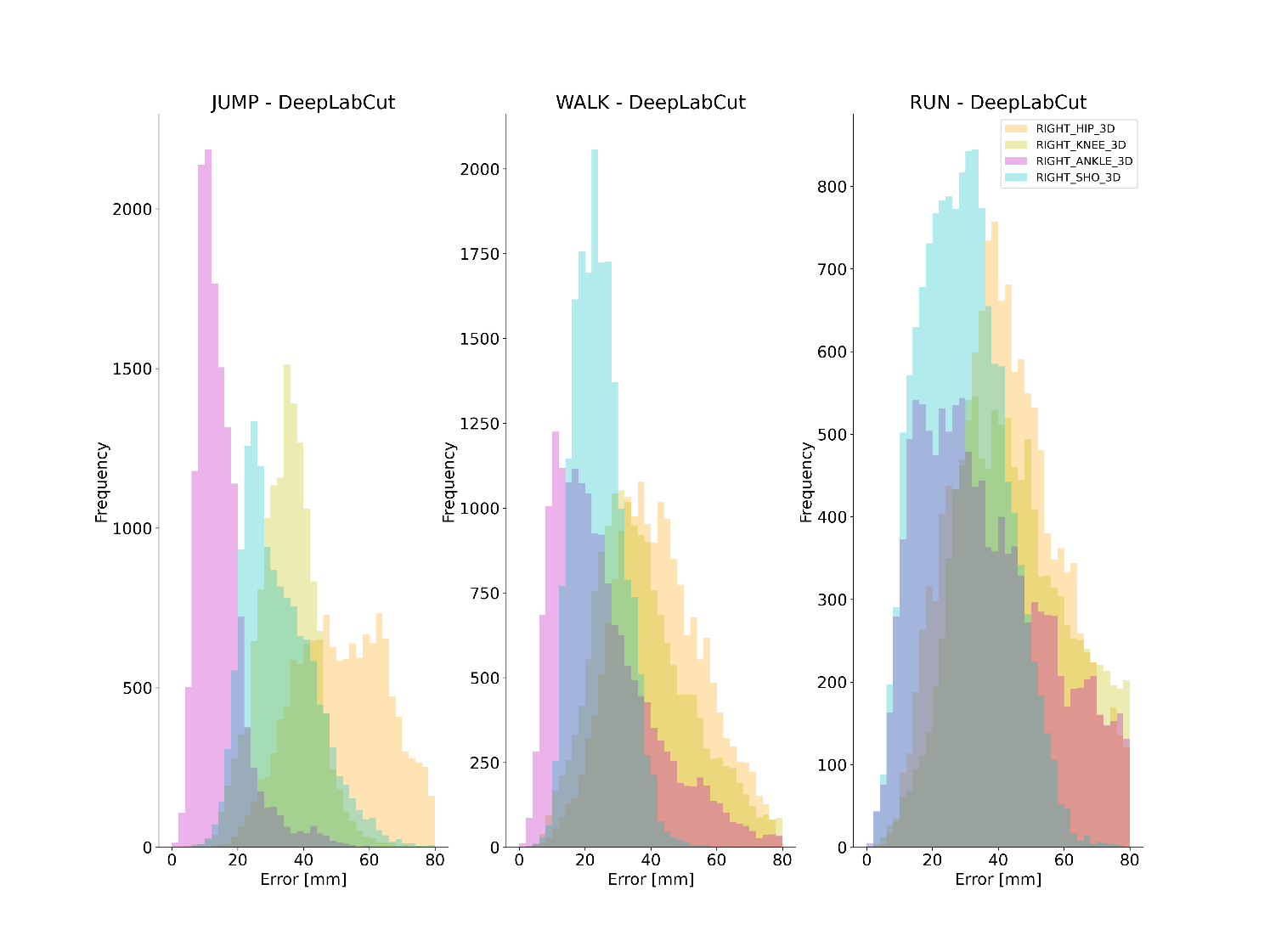


**Figure A12. Error distributions for DeepLabCut at the right shoulder, hip, knee and ankle joint centres for jumping (left), walking (centre) and running (right).**

**Table A2. Bland-Altman analysis results of X, Y and Z axis differences during walking. OpenPose – OP, AlphaPose – AP, DeepLabCut – DLC.**

| **WALK** | **Mean Difference (Bias) (mm)** | | | **Random Error (± SD of Bias)** | | | **95% LoA (Lower - Upper Limit)** | | | | | |
| --- | --- | --- | --- | --- | --- | --- | --- | --- | --- | --- | --- | --- |
|  | **OP** | **AP** | **DLC** | **OP** | **AP** | **DLC** | **OP** | | **AP** | | **DLC** | |
| **Shoulder X** | 16 | 17 | 1 | 9 | 9 | 8 | -2 | 34 | 0 | 34 | -14 | 16 |
| **Shoulder Y** | 10 | 2 | 2 | 13 | 14 | 13 | -15 | 35 | -25 | 29 | -23 | 27 |
| **Shoulder Z** | 15 | 18 | 19 | 9 | 9 | 8 | -2 | 33 | 1 | 35 | 3 | 35 |
| **Hip X** | -22 | -22 | -18 | 10 | 10 | 10 | -41 | -3 | -41 | -2 | -38 | 3 |
| **Hip Y** | 17 | 7 | 17 | 13 | 13 | 18 | -9 | 42 | -18 | 32 | -18 | 51 |
| **Hip Z** | 8 | -6 | -22 | 15 | 14 | 23 | -21 | 37 | -34 | 22 | -66 | 22 |
| **Knee X** | -4 | -6 | -4 | 7 | 4 | 9 | -17 | 9 | -14 | 3 | -22 | 13 |
| **Knee Y** | 5 | -3 | 7 | 17 | 20 | 35 | -28 | 38 | -42 | 36 | -61 | 76 |
| **Knee Z** | -23 | -24 | -28 | 12 | 12 | 16 | -46 | 0 | -47 | 0 | -60 | 4 |
| **Ankle X** | -4 | -3 | -5 | 5 | 5 | 13 | -15 | 6 | -12 | 6 | -30 | 19 |
| **Ankle Y** | -1 | -10 | -8 | 12 | 16 | 44 | -25 | 22 | -42 | 22 | -93 | 78 |
| **Ankle Z** | 5 | 4 | -11 | 10 | 11 | 13 | -15 | 25 | -18 | 27 | -36 | 14 |

**Table A3. Linear regression analysis results of X, Y and Z axis differences during walking. OpenPose – OP, AlphaPose – AP, DeepLabCut – DLC.**

| **WALK** | **R2** | | | **Gradient** | | | **Intercept** | | |
| --- | --- | --- | --- | --- | --- | --- | --- | --- | --- |
|  | **OP** | **AP** | **DLC** | **OP** | **AP** | **DLC** | **OP** | **AP** | **DLC** |
| **Shoulder X** | 0.99 | 0.99 | 0.99 | 0.97 | 0.97 | 0.98 | -10 | -11 | 3 |
| **Shoulder Y** | 1.00 | 1.00 | 1.00 | 1.00 | 1.00 | 1.00 | -10 | -2 | -3 |
| **Shoulder Z** | 0.99 | 0.99 | 0.99 | 0.97 | 0.97 | 0.98 | 22 | 22 | 6 |
| **Hip X** | 0.98 | 0.98 | 0.98 | 1.00 | 1.00 | 0.98 | 22 | 22 | 20 |
| **Hip Y** | 1.00 | 1.00 | 1.00 | 1.00 | 1.00 | 1.00 | -16 | -7 | -17 |
| **Hip Z** | 0.96 | 0.96 | 0.93 | 0.83 | 0.84 | 0.69 | 142 | 147 | 303 |
| **Knee X** | 0.99 | 1.00 | 0.99 | 0.99 | 0.99 | 1.00 | 5 | 6 | 5 |
| **Knee Y** | 1.00 | 1.00 | 1.00 | 1.00 | 1.00 | 1.01 | -4 | 4 | -5 |
| **Knee Z** | 0.91 | 0.91 | 0.83 | 0.87 | 0.88 | 0.79 | 85 | 81 | 131 |
| **Ankle X** | 0.99 | 1.00 | 0.97 | 1.00 | 1.00 | 0.97 | 4 | 4 | 7 |
| **Ankle Y** | 1.00 | 1.00 | 1.00 | 1.01 | 1.01 | 1.00 | 4 | 13 | 8 |
| **Ankle Z** | 0.96 | 0.96 | 0.94 | 1.04 | 1.07 | 0.91 | -10 | -14 | 24 |

**Table A4. Bland-Altman analysis results of X, Y and Z axis differences during running. OpenPose – OP, AlphaPose – AP, DeepLabCut – DLC.**

| **RUN** | **Mean Difference (Bias) (mm)** | | | **Random Error (± SD of Bias)** | | | **95% LoA (Lower - Upper Limit)** | | | | | |
| --- | --- | --- | --- | --- | --- | --- | --- | --- | --- | --- | --- | --- |
|  | **OP** | **AP** | **DLC** | **OP** | **AP** | **DLC** | **OP** | | **AP** | | **DLC** | |
| **Shoulder X** | 12 | 11 | -4 | 12 | 10 | 11 | -12 | 35 | -10 | 31 | -25 | 18 |
| **Shoulder Y** | 1 | -12 | -16 | 16 | 17 | 15 | -30 | 32 | -45 | 21 | -45 | 13 |
| **Shoulder Z** | 14 | 17 | 17 | 10 | 12 | 11 | -6 | 34 | -6 | 40 | -6 | 39 |
| **Hip X** | -18 | -17 | -15 | 10 | 9 | 17 | -37 | 1 | -35 | 1 | -47 | 18 |
| **Hip Y** | 5 | -10 | -3 | 15 | 15 | 20 | -24 | 34 | -39 | 19 | -42 | 36 |
| **Hip Z** | -5 | -10 | -31 | 15 | 17 | 24 | -35 | 25 | -44 | 23 | -78 | 16 |
| **Knee X** | -5 | -6 | -4 | 7 | 6 | 14 | -18 | 9 | -17 | 5 | -32 | 23 |
| **Knee Y** | -18 | -32 | -26 | 24 | 29 | 49 | -65 | 28 | -88 | 25 | -122 | 70 |
| **Knee Z** | -30 | -28 | -34 | 12 | 15 | 17 | -53 | -7 | -57 | 1 | -67 | -1 |
| **Ankle X** | -2 | -2 | -3 | 7 | 6 | 26 | -15 | 12 | -13 | 9 | -54 | 48 |
| **Ankle Y** | -14 | -31 | -23 | 18 | 26 | 69 | -49 | 21 | -82 | 20 | -157 | 111 |
| **Ankle Z** | 2 | 3 | -7 | 12 | 15 | 38 | -22 | 25 | -26 | 32 | -81 | 67 |

**Table A5. Linear regression analysis results of X, Y and Z axis differences during running. OpenPose – OP, AlphaPose – AP, DeepLabCut – DLC.**

| **RUN** | **R^2^** | | | **Gradient** | | | **Intercept** | | |
| --- | --- | --- | --- | --- | --- | --- | --- | --- | --- |
|  | **OP** | **AP** | **DLC** | **OP** | **AP** | **DLC** | **OP** | **AP** | **DLC** |
| **Shoulder X** | 0.98 | 0.99 | 0.99 | 0.95 | 0.96 | 0.96 | -3 | -4 | 10 |
| **Shoulder Y** | 0.99 | 1.00 | 1.00 | 0.99 | 1.00 | 1.00 | -5 | 12 | 16 |
| **Shoulder Z** | 0.74 | 0.99 | 0.99 | 0.96 | 0.96 | 0.96 | 35 | 40 | 44 |
| **Hip X** | 0.97 | 0.97 | 0.95 | 0.97 | 0.98 | 0.97 | 21 | 19 | 17 |
| **Hip Y** | 0.98 | 0.98 | 0.98 | 0.99 | 1.00 | 0.99 | -15 | 4 | -4 |
| **Hip Z** | 0.54 | 0.70 | 0.65 | 0.67 | 0.64 | 0.59 | 303 | 340 | 400 |
| **Knee X** | 0.98 | 0.98 | 0.96 | 1.00 | 1.00 | 0.99 | 6 | 7 | 6 |
| **Knee Y** | 0.97 | 0.98 | 0.98 | 0.99 | 1.00 | 1.00 | 6 | 23 | 16 |
| **Knee Z** | 0.63 | 0.72 | 0.71 | 0.71 | 0.69 | 0.64 | 173 | 179 | 211 |
| **Ankle X** | 0.99 | 0.99 | 0.92 | 1.00 | 1.00 | 1.00 | 2 | 2 | 3 |
| **Ankle Y** | 0.99 | 0.99 | 0.99 | 1.00 | 1.01 | 1.00 | 12 | 36 | 22 |
| **Ankle Z** | 0.99 | 0.98 | 0.88 | 1.02 | 1.02 | 0.86 | -6 | -8 | 39 |

**Table A6. Bland-Altman analysis results of X, Y and Z axis differences during jumping. OpenPose – OP, AlphaPose – AP, DeepLabCut – DLC.**

| **JUMP** | **Mean Difference (Bias) (mm)** | | | **Random Error (± SD of Bias)** | | | **95% LoA (Lower - Upper Limit)** | | | | | |
| --- | --- | --- | --- | --- | --- | --- | --- | --- | --- | --- | --- | --- |
|  | **OP** | **AP** | **DLC** | **OP** | **AP** | **DLC** | **OP** | | **AP** | | **DLC** | |
| **Shoulder X** | 6 | 3 | -11 | 13 | 13 | 13 | -20 | 32 | -23 | 28 | -37 | 15 |
| **Shoulder Y** | 18 | 20 | 16 | 16 | 17 | 16 | -15 | 50 | -13 | 52 | -15 | 47 |
| **Shoulder Z** | 11 | 11 | 11 | 16 | 18 | 17 | -21 | 43 | -24 | 46 | -22 | 44 |
| **Hip X** | -25 | -23 | -17 | 10 | 9 | 11 | -45 | -6 | -41 | -5 | -38 | 3 |
| **Hip Y** | 16 | 10 | 29 | 14 | 16 | 22 | -11 | 43 | -22 | 41 | -16 | 73 |
| **Hip Z** | -6 | -12 | -27 | 14 | 15 | 23 | -34 | 22 | -42 | 18 | -73 | 18 |
| **Knee X** | -1 | -2 | -2 | 6 | 4 | 5 | -12 | 11 | -11 | 6 | -11 | 7 |
| **Knee Y** | 6 | 5 | 10 | 9 | 9 | 13 | -12 | 25 | -12 | 22 | -15 | 36 |
| **Knee Z** | -25 | -25 | -28 | 11 | 10 | 14 | -46 | -4 | -44 | -5 | -56 | -1 |
| **Ankle X** | -5 | -4 | -5 | 5 | 3 | 4 | -15 | 4 | -11 | 3 | -12 | 3 |
| **Ankle Y** | 2 | -3 | 0 | 7 | 6 | 9 | -11 | 15 | -15 | 9 | -17 | 17 |
| **Ankle Z** | 9 | 9 | -8 | 7 | 9 | 10 | -5 | 24 | -9 | 27 | -27 | 11 |

**Table A7. Linear regression analysis results of X, Y and Z axis differences during jumping. OpenPose – OP, AlphaPose – AP, DeepLabCut – DLC.**

| **JUMP** | **R^2^** | | | **Gradient** | | | **Intercept** | | |
| --- | --- | --- | --- | --- | --- | --- | --- | --- | --- |
|  | **OP** | **AP** | **DLC** | **OP** | **AP** | **DLC** | **OP** | **AP** | **DLC** |
| **Shoulder X** | 0.77 | 0.78 | 0.77 | 0.81 | 0.84 | 0.85 | 24 | 24 | 36 |
| **Shoulder Y** | 0.98 | 0.98 | 0.98 | 0.90 | 0.90 | 0.91 | -56 | -58 | -51 |
| **Shoulder Z** | 1.00 | 1.00 | 1.00 | 0.95 | 0.94 | 0.95 | 63 | 67 | 59 |
| **Hip X** | 0.83 | 0.85 | 0.81 | 0.95 | 0.94 | 0.96 | 30 | 29 | 20 |
| **Hip Y** | 0.98 | 0.97 | 0.93 | 0.92 | 0.90 | 0.93 | -56 | -63 | -64 |
| **Hip Z** | 0.99 | 0.99 | 0.98 | 0.97 | 0.96 | 0.97 | 33 | 44 | 58 |
| **Knee X** | 0.97 | 0.98 | 0.98 | 0.97 | 0.97 | 0.99 | 5 | 6 | 4 |
| **Knee Y** | 0.99 | 0.99 | 0.98 | 1.01 | 1.00 | 1.03 | 0 | -5 | 5 |
| **Knee Z** | 0.99 | 0.99 | 0.99 | 0.98 | 0.98 | 0.96 | 36 | 33 | 48 |
| **Ankle X** | 0.98 | 0.99 | 0.99 | 0.99 | 0.98 | 0.98 | 7 | 8 | 7 |
| **Ankle Y** | 0.99 | 0.99 | 0.98 | 0.98 | 0.98 | 0.98 | -13 | -6 | -13 |
| **Ankle Z** | 1.00 | 0.99 | 0.99 | 1.01 | 1.00 | 1.02 | -11 | -8 | 6 |
